# Impacts of climatic factors on the global spatiotemporal distribution patterns of *Xylobolus subpileatus* (Stereaceae, Russulales)

**DOI:** 10.3897/mycokeys.133.190063

**Published:** 2026-05-28

**Authors:** Yunlin Xu, Anyi Niu, Jiaxi Cao, Rong Fan, Shuanghui He

**Affiliations:** 1 Jiangsu Key Laboratory of Coal-based Greenhouse Gas Control and Utilization, China University of Mining and Technology, Xuzhou, 221008, China College of New Energy and Environment, Jilin University Changchun China; 2 Carbon Neutrality Institute, China University of Mining and Technology, Xuzhou, 221008, China Carbon Neutrality Institute, China University of Mining and Technology Xuzhou China; 3 College of New Energy and Environment, Jilin University, Changchun, 130012, China Jiangsu Key Laboratory of Coal-based Greenhouse Gas Control and Utilization, China University of Mining and Technology Xuzhou China; 4 School of Ecology and Nature Conservation, Beijing Forestry University, Beijing 100083, China School of Ecology and Nature Conservation, Beijing Forestry University Beijing China

**Keywords:** Climate factor, conservation, maximum entropy, potential distribution area, *Xylobolus subpileatus*

## Abstract

*Xylobolus subpileatus*, a corticioid fungus associated with white rot in woody angiosperms, represents a valuable resource for pharmaceutical and industrial applications due to its production of antitumor and neuroprotective compounds. It plays a significant ecological role in lignin decomposition and the global carbon cycle. Assessing the impact of climate change on its distribution is essential for germplasm conservation. This study employed the MaxEnt model with CMIP6 climate data to project potential suitable habitats across several periods: the Mid Holocene, the present (1970–2000), and future decades (2030s–2090s). The model performed reliably, with current predictions aligning well with known occurrences. Annual precipitation, precipitation of the driest quarter, and annual mean temperature were identified as the dominant factors shaping its distribution. The total suitable area under current conditions is approximately 2944.21 × 10^4^ km^2^, categorized into low, moderate, and high suitability zones, with the most highly suitable regions concentrated in North America. Over time, the suitable area showed an initial expansion followed by a contraction. These projections help identify previously unexplored potential habitats and provide a scientific basis for the conservation and sustainable management of this species.

## Introduction

*Xylobolus subpileatus* (Berk. & M.A. Curtis) Boidin is a corticioid fungus belonging to the family Stereaceae within the order Russulales (Yuan et al. 2026). It possesses an effuso-reflexed, perennial basidiome. It is primarily found in the southeastern United States, with fewer records of distribution in other regions, particularly in China. White-rot fungi, such as *X. subpileatus*, are known to cause white rot in certain woody angiosperms (Lentz, 1956). This functional group plays a crucial role in the global carbon cycle (Dong et al. 2025; Zhao et al. 2025, 2026; Zhang et al. 2026; Cao and Zhang 2026). Specifically, *X. subpileatus* enhances forest floor heterogeneity by creating distinct decay patterns in wood. These patterns provide ecological niches for other organisms, making this species a key component of old-growth forest ecosystems.

Research has shown that *Xylobolus subpileatus* has high scientific and translational value due to its diverse bioactive compounds. Its secondary metabolites include industrial chemical intermediates, potent broad-spectrum antitumor lead compounds, and selective neuroprotective agents that have proven effective in preclinical studies (Tian 2016; Felegyi et al. 2023). For example, ten metabolites were isolated from the fermentation broth and methanol extract of the fungal mycelium from *X. subpileatus* (Tian 2016). Among these compounds, seven were structurally confirmed, and six were identified as known substances. One of these known substances was 4-hydroxybenzaldehyde, which is a major chemical constituent of *Gastrodia elata* Bl. Another compound, stella sterol, a naturally occurring sterol, has been shown to inhibit the proliferation of K562 tumor cells at specific concentrations (Feng et al. 2015). Ergosterol peroxide, also identified in the extract, is a steroid derivative belonging to the ergosterol class. It is commonly found in fungi, yeasts, lichens, and marine sponges. Despite their ecological and economic importance, *X. subpileatus* remains poorly studied in terms of their large-scale spatial and temporal distribution. This knowledge gap is largely due to its unique life cycles and complex growth form. Current information on their natural habitats and occurrence patterns is limited, apart from a few geographically restricted records. Therefore, it is imperative to develop robust conservation and restoration strategies. These efforts are essential for the protection and sustainable utilization of these ecologically significant fungal species.

Species Distribution Models (SDMs) are practical tools that estimate the potential geographic distributions of species. These models are developed based on observed species occurrence data and environmental variables or spatial characteristics of potential habitats (Franklin 2010). Geographic Information System (GIS) technology has transformed how we understand spatial phenomena (Peterson et al. 2003; Graham et al. 2006; Guo et al. 2019a, 2019b; Wan et al. 2021). The biological sciences have accumulated a vast repository of species occurrence records over more than a century. These datasets, stored in numerous biodiversity databases, represent an invaluable resource for ecological modeling. Species occurrence data generally fall into two categories: presence-only data and presence–absence data (Elith et al. 2011). Environmental variables used in SDMs can be broadly classified into two types. Biological variables include factors such as parasitism, predation, and dispersal ability. Non-biological variables typically refer to abiotic factors, such as climate and soil characteristics. SDMs are constructed by applying either statistical or machine-learning algorithms to a training dataset. This dataset consists of known species occurrence points and associated environmental variables. After considering the respective strengths and limitations of each statistical method, we have identified the Maximum Entropy (MaxEnt) model as the most appropriate approach for predicting the distribution of the target species. This conclusion is drawn based on a comprehensive evaluation of multiple modeling methodologies.

The Maximum Entropy model (MaxEnt) has been widely used in species distribution modeling, including ecology, evolutionary biology, conservation, and biosecurity (Phillips et al. 2006; Phillips and Dudík 2008; Elith and Leathwick 2009; Anderson 2013; Guo et al. 2017; Wang and Guan 2023). MaxEnt is often the preferred modeling approach for several reasons. It offers an accessible and user-friendly interface. The model is computationally efficient, easy to operate, and performs well with relatively small sample sizes. In addition, it demonstrates strong predictive performance across a range of ecological contexts (Phillips et al. 2006; Merow et al. 2013). At present, MaxEnt has become the dominant method for predicting species distributions in fungi, animals and plants (Yuan et al. 2015; Banasiak et al. 2019; Guo et al. 2019a, 2019b; Qin et al. 2020; Bie et al. 2021; Wei et al. 2021; Yu et al. 2021; Chen et al. 2022; Zhan et al. 2022; Cai et al. 2023). Globally, the MaxEnt model has been extensively employed in research on species distribution and variation (Zwiener and Alves 2023). Following a thorough evaluation of the MaxEnt model’s advantages and its suitability for addressing our research objectives, we selected this method to predict the potential distribution of *X. subpileatus*. We are confident that the application of this model will yield valuable insights and contribute to a more comprehensive understanding of the species’ distributional patterns.

To date, most existing studies on *X. subpileatus* have concentrated on its taxonomy and phylogenetic relationships (Bernicchia and Gorjón 2010; Vu et al. 2019). However, despite the ecological importance of this species, little attention has been given to predicting its distribution using modeling approaches. In particular, the MaxEnt model has not yet been applied to assess the distribution patterns of corticioid fungi. To address this research gap, the present study aimed to explore the potential distribution of *X. subpileatus* using the MaxEnt modeling framework in combination with environmental variables. This study had two specific objectives. First, we estimated the potential suitable habitat range of *X. subpileatus* under different climatic conditions and identified the key climatic variables influencing its global distribution. Second, we predicted the spatial distribution of suitable areas for *X. subpileatus* across the Mid-Holocene, the present, and four future scenarios, and analyzed temporal shifts in suitable habitat distributions across these periods.

## Materials and methods

### Biological characteristics of the species

The basidiome is effuse-reflexed, with a coriaceous to corky texture. The hymenophore is greyish to brown in color and becomes tuberculate with age. This species commonly occurs on *Quercus* spp., but it has also been found on *Ostrya carpinifolia* Scop., and in one instance, on a railway sleeper (Bernicchia and Gorjón 2010). The probability of fruiting of *X. subpileatus* significantly increases during the process of wood decomposition to reach its maximum in the oldest gaps, approximately 40 years after treefall (Taudiere et al. 2017). The compounds isolated from this species exhibit pronounced biological activity and substantial therapeutic potential. As secondary metabolites, they have dual functional significance. First, they serve as structurally diverse and synthetically tractable chemical intermediates for medicinal chemistry optimization. Second, they function as pharmacologically privileged scaffolds with validated target engagement. This is evidenced by potent, selective cytotoxicity against multiple human cancer cell lines at pharmacologically relevant concentrations and by robust, mechanism-informed neuroprotective effects in established cellular and preclinical models. Collectively, these properties position them as high-value leads for oncology and neurodegeneration drug discovery.

### Species occurrence records

A comprehensive dataset comprising 398 geographic occurrence records of *X. subpileatus* was obtained from the Global Biodiversity Information Facility (GBIF; https://www.gbif.org; accessed on December 2, 2021). Records that were duplicated or lacked geographic coordinate information (i.e., missing longitude or latitude) were excluded from the dataset. To further refine the data, ENMTools (Warren et al. 2010) was employed. This software eliminates redundant records occurring within the same spatial grid cell, as opposed to relying on distance-based filtering methods. Following data cleaning and refinement, a total of 232 unique distribution points were retained for the development of the MaxEnt model (see Suppl. material 1: fig. S1). In accordance with MaxEnt input requirements, the final distribution dataset was saved in comma-separated values (CSV) format.

### Climatic data and environmental variables

The dataset included a total of 21 environmental variables, comprising 19 climatic variables and 2 topographic variables, viz. aspect and slope. All variables were obtained from the WorldClim database (http://www.worldclim.org). The climatic data were derived from the BCC-CSM2-MR general circulation model (GCM), collected on a monthly basis. These data were then mapped onto a spatial grid with a resolution of 2.5 arc-minutes, which corresponds to approximately 5 km × 5 km. Topographic variables were sourced from the same dataset. The mapping boundaries were defined based on the Global Administrative Unit Layers (Stimson 2023). The selected variables included aspect, cumulative monthly precipitation, and average monthly temperatures (both minimum and maximum values). In addition, 20 bioclimatic parameters derived from climate data were used to reflect biologically relevant variations in environmental conditions (Fan et al. 2022). For the MaxEnt modeling, 20 climate variables were selected for different temporal scenarios. These included the Middle-Holocene, the current period (1970–2000), and four future time slices: 2021–2040, 2041–2060, 2061–2080, and 2081–2100. To simulate future climate scenarios, the study adopted the SSP2-4.5 pathway. This Shared Socioeconomic Pathway (SSP) reflects a “middle of the road” scenario, representing moderate challenges to both climate mitigation and adaptation. Initially, all environmental variables were provided in raster format. These files were subsequently processed using ArcGIS 10.8, including clipping and resampling procedures. The final outputs were stored in ASCII format for compatibility with the MaxEnt model.

### Data Preparation

Pearson correlation coefficient (r) is a widely used metric for quantifying the strength of linear relationships between two variables. The absolute value of *r* (|*r*|) ranges from 0 to 1, with higher values indicating stronger correlations. To improve the predictive performance of the MaxEnt model, Pearson correlation analysis was applied to all environmental variables (Fotheringham and Oshan 2016). In this study, bivariate correlation analysis was conducted using SPSS software (version 26.0; https://spss.en.softonic.com/). This analysis aimed to evaluate the relationships between ecological variables and to support the development of regression models. For each pair of significantly correlated variables, only the variable with the higher contribution to the MaxEnt model was retained (see Suppl. material 1: fig. S2). Variables were considered significantly correlated if the absolute value of their correlation coefficient exceeded 0.80 (Wisz et al. 2013; Zeng et al. 2016). Ultimately, from each highly correlated pair, only the variable contributing more substantially to the model was included in further analysis. The data analysis was conducted using DMSAS 1.9.

### MaxEnt analysis

The MaxEnt model (version 3.4.1) was employed to predict the potential distribution of the species. Two key parameters in the MaxEnt algorithm—the regularization multiplier (RM) and feature classes (FC)—were adjusted to balance model fit and complexity, and to define the types of constraints incorporated in the model. The Akaike Information Criterion (AIC) was used to evaluate the trade-off between model fit and complexity. It is considered a robust criterion for assessing model performance (Phillips and Dudík 2008). Under the default settings (RM = 1.0, FC = LQHP), the model exhibited insufficient fit. In contrast, the optimized configuration (RM = 3.0, FC = LQHPT) resulted in the lowest delta AIC value, which was zero, indicating the best model performance. Other model parameters were configured as follows: 75% of the occurrence records were used as the training set, while the remaining 25% were used as the test set. Cross-validation was selected as the replicated run type. The maximum number of iterations was set to 1000. The importance of each ecological variable in determining habitat suitability was evaluated using the Jackknife test. Additionally, model performance was assessed by constructing a receiver operating characteristic (ROC) curve. Based on previous research, habitat suitability thresholds were defined using the habitat suitability index (HSI). The categories were as follows: 0–0.1, unsuitable region; 0.1–0.3, low-suitability region; 0.3–0.5, moderate-suitability region; and 0.5–1.0, high-suitability region (Guo et al. 2019a; Chen et al. 2022).

Two metrics were used to evaluate predictive accuracy: the area under the curve (AUC) and the true skill statistic (TSS). Both indices range from 0 to 1, with higher values indicating better predictive performance. All remaining model options were left at their default settings. Model performance, as indicated by AUC values, was categorized into five levels: failure (0.5–0.6), low accuracy (0.6–0.7), moderate accuracy (0.7–0.8), high accuracy (0.8–0.9), and very high accuracy (0.9–1.0) (Pearce and Ferrier 2000). The reliability of the model increases as the absolute difference between the training AUC and test AUC values decreases (Warren and Seifert 2011). Finally, based on the regression model, MaxEnt generated a probability distribution map for *X. subpileatus*.

### Exploration of alterations in spatial pattern

Spatial pattern refers to the degree and structure of spatial variation in ecosystem attributes. Changes in spatial patterns can reflect shifts in ecological processes and biodiversity. Understanding such changes is crucial for effective species management and conservation planning (Zurell et al. 2020; Wu et al. 2021; Zhao et al. 2022). In this study, the **SDMtoolbox** toolkit was employed for post-processing the results of the species distribution model. A binary suitability map was generated by applying a threshold determined using the equal interval method. This approach enables the identification of areas where the species’ distribution is expected to expand, remain stable, or contract (Brown 2014). To evaluate spatial shifts in suitable habitat, the **Centroid Change Tool** within SDMtoolbox was utilized. This tool analyzes changes in the centroid of suitable habitat under current and projected future climate scenarios. It provides key spatial metrics, including migration direction and distance of the habitat centroid. All spatial analyses and visualizations were performed using **ArcGIS 10.8**. Subsequent analysis and map-based visualization were also conducted within this software environment.

## Results

### Model accuracy analysis and suitable area division

**Figure 1.**
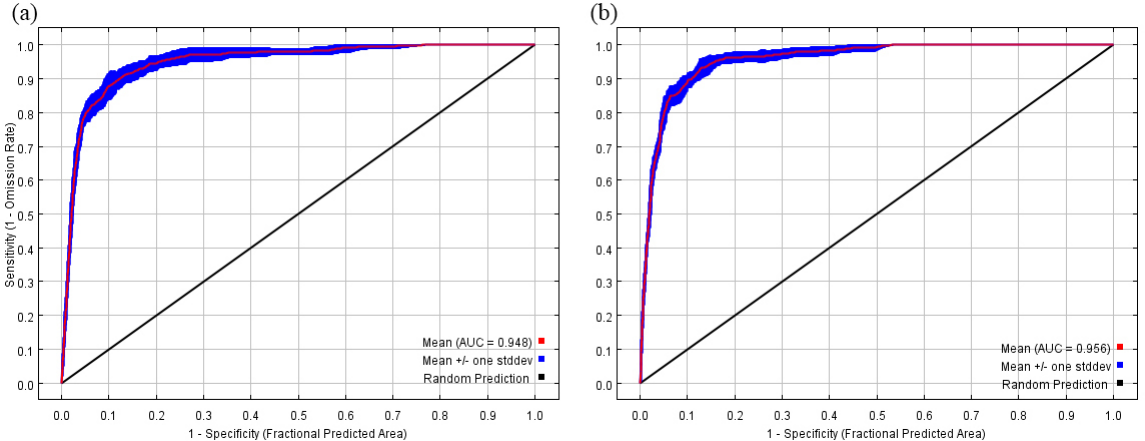
ROC curve of the MaxEnt model created for *X. subpileatus*. **a**. Mid Holocene; **b**. Current.

The receiver operating characteristic (ROC) curve illustrates the averaged results of replicate runs based on the same environmental dataset (Fig. 1). For the historical and current distribution models, the training AUC values (AUC_TRAIN_) ranged from 0.957 ± 0.013 to 0.963 ± 0.010. The test AUC values (AUC_TEST_) were 0.948 ± 0.009 and 0.956 ± 0.007, respectively. The absolute differences between AUC_TRAIN_ and AUC_TEST_ (|AUC_DIFF_|) were less than 0.01, indicating excellent model stability and reliability. The models predicting future potential distributions also achieved high AUC values, all exceeding 0.946. Furthermore, the mean true skill statistic (TSS) reached 0.886, demonstrating strong predictive performance. Compared with previous studies in the same field, this study yielded exceptionally high AUC and TSS values, underscoring the significant methodological improvements and contributions to species distribution modeling. The ROC curve serves as a valuable tool for evaluating model accuracy. Higher AUC and TSS values indicate a stronger ability to correctly identify species presence and absence. Overall, the results strongly support the high accuracy and reliability of the MaxEnt model predictions in this study.

### Contribution analysis of environmental variables

To construct the historical model, the optimized MaxEnt model incorporated twelve key bioclimatic factors, including variables related to temperature and precipitation (Table 1). By integrating these comprehensive factors, the model provides accurate predictions and effectively identifies potential threats to species survival. Overall, the MaxEnt model proves to be a highly effective tool for studying species distribution patterns. It utilizes iterative algorithms to refine evaluation factors and offers detailed analyses of both historical and future distribution scenarios. This capability makes it particularly valuable for developing conservation strategies aimed at protecting vulnerable species from various environmental threats.

**Table 1.** Bioclimatic factors used for the final model development and their contributions.

Variable	Description/Unit	Contribution (%)					
		MID	Current	2030s	2050s	2070s	2090s
**Bio1**	Annual Mean Temperature/°C	31.1	30.6	38.8	39.4	36.8	31.1
**Bio2**	Mean Diurnal Range (Mean of monthly (max temp – min temp))/°C	2	2	2	1.8	1.7	2
**Bio3**	Isothermality (Bio2/Bio7) (×100)%	2.7	–	1.5	–	–	2.7
**Bio4**	Temperature Seasonality (standard deviation × 100)/°C	–	9.1	–	–	8.8	–
**Bio5**	Max Temperature of Warmest Month/°C	–	–	–	–	–	–
**Bio6**	Min Temperature of Coldest Month/°C	–	–	–	–	–	–
**Bio7**	Temperature Annual Range (Bio5-Bio6)/°C	–	–	–	–	–	–
**Bio8**	Mean Temperature of Wettest Quarter/°C	2.8	0.8	0.8	–	0.1	2.8
**Bio9**	Mean Temperature of Driest Quarter/°C	–	0.3	0.2	0.4	0.3	–
**Bio10**	Mean Temperature of Warmest Quarter/°C	0.8	0.1	0.2	0.3	0.2	0.8
**Bio11**	Mean Temperature of Coldest Quarter/°C	–	–	–	4.6	–	–
**Bio12**	Annual Precipitation/mm	32.5	29.4	29.6	33.3	31.5	32.5
**Bio13**	Precipitation of Wettest Month/mm	–	–	–	–	–	–
**Bio14**	Precipitation of Driest Month/mm	–	–	–	–	–	–
**Bio15**	Precipitation Seasonality (Coefficient of Variation)/%	0.9	–	–	–	–	0.9
**Bio16**	Precipitation of Wettest Quarter/mm	–	–	1.9	2.4	1.7	–
**Bio17**	Precipitation of Driest Quarter/mm	11.6	10.1	2.5	6.4	10.5	11.6
**Bio18**	Precipitation of Warmest Quarter/mm	7.3	8.1	12.1	4.5	3.6	7.3
**Bio19**	Precipitation of Coldest Quarter/mm	8.4	4.7	10.4	6.9	4.9	8.4
**Elevation**	aspect/–	–	0.6	–	–	–	–
**Elevation**	slope/–	–	4.1	–	–	–	–

The dash (–) means that the environmental variable is not included for modeling the geographic distribution due to autocorrelation.

**Figure 2.**
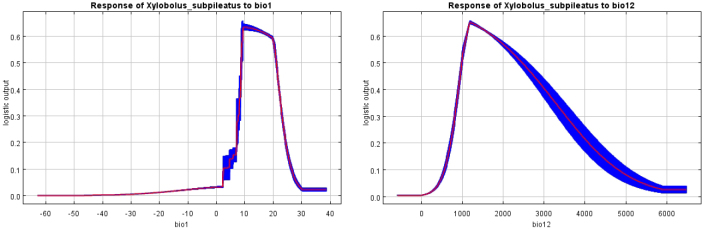
Response curves of main environmental factors for the probability of current of *X. subpileatus*.

The results of the jackknife test assessing variable importance are presented in Suppl. material 1: fig. S3. This suggests that bio4 contains unique information that cannot be substituted by other variables. By combining the results of environmental variable analysis and detection metrics, we conclude that the dominant factors influencing the distribution of *X. subpileatus* are annual mean temperature (bio1) and annual precipitation (bio12). The response curves of these two key variables, shown in Fig. 2, illustrate their influence on model predictions and clarify the relationship between habitat suitability, species occurrence probability, and the threshold ranges of relevant ecological factors.

### Mid Holocene potential distribution

The original output of the MaxEnt model was reclassified, and the area of each suitable habitat category was calculated using ArcGIS 10.8 (Table 2). Statistical results showed that, during the Mid-Holocene period, the potential suitable habitats of *X. subpileatus* covered a total area of 2,797.05 × 10^4^ km^2^.

**Table 2.** Predicted suitable areas under past, current and future climatic conditions (× 10^4^ km^2^).

Decades	Total suitable region	Lowly suitable region	Moderately suitable region	Highly suitable region
**MID**	2797.05	1885.59	515.85	395.61
**Current**	2944.21	2067.48	520.03	356.7
**2021–2040**	3531.91	2328.12	712.04	491.75
**2041–2060**	3385.73	2183.22	724.38	478.13
**2061–2080**	3642.36	2305.65	781.64	555.07
**2081–2100**	3117.47	2013.69	720.6	383.18

**Figure 3.**
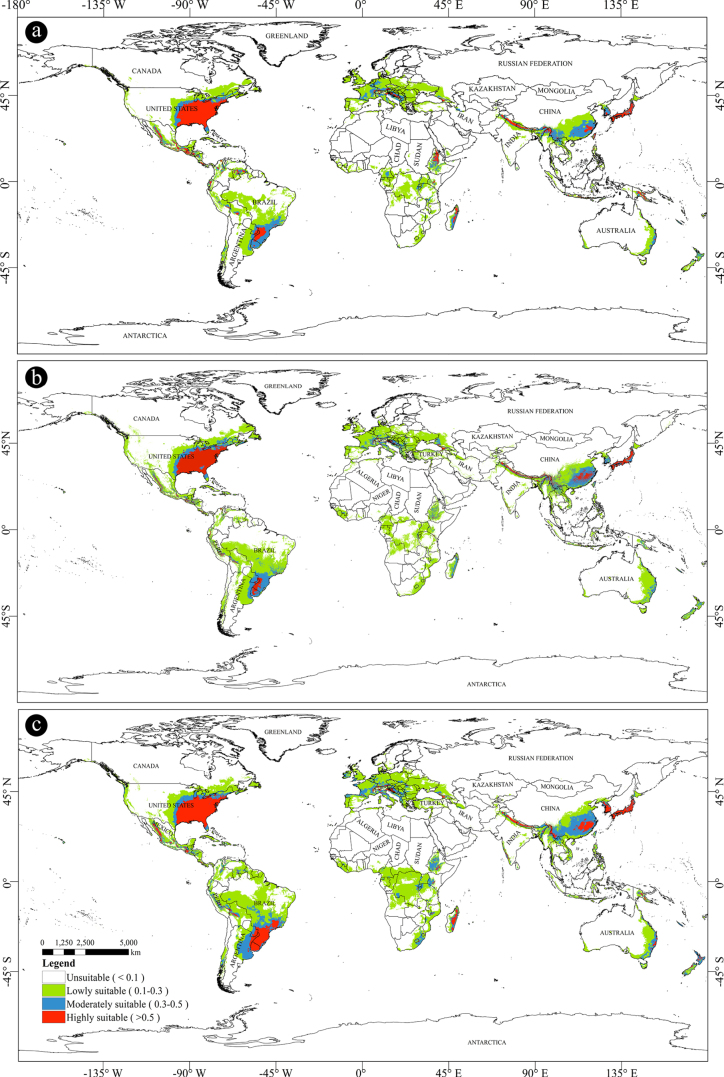
Predicted distribution of *X. subpileatus*. **a**. Mid Holocene; **b**. Current (1970–2000); **c**. 2030s (2021–2040). The source of map: Stimson 2023.

A suitable habitat distribution map for the Mid-Holocene is presented in Fig. 3a. Among the predicted areas, highly suitable regions accounted for 14.14% of the total suitable area, while moderately suitable regions represented 18.44%. Under the Mid-Holocene climate scenario, suitable habitats for *X. subpileatus* were concentrated mainly in North America and South America, which contributed 22.95% and 21.82% of the total suitable area, respectively (Fig. 3). Detailed data on the potential habitat areas for each continent are provided in Suppl. material 1: table SS1. These results offer important insights into the distinct ecological characteristics of different regions and provide valuable references for promoting biodiversity conservation and sustainable ecosystem management.

### Current potential distribution

The current suitable habitat distribution map for *X. subpileatus* is shown in Fig. 3b. Statistical analysis revealed that the current potential suitable regions cover a total area of 2,944.21 × 10^4^ km^2^. Among these, highly suitable regions account for 12.12% of the total suitable area, while moderately suitable regions represent 17.66%. Compared with the Mid-Holocene scenario, the overall extent of potential suitable habitats has slightly increased. However, the area classified as highly suitable shows a marginal decline. This increase in overall habitat suitability is unevenly distributed across regions, with North America and Asia representing the primary clusters of suitable areas. Regional differences in climatic conditions, topographical features, and ecological niches likely contributed to the observed patterns. These environmental factors may either facilitate or restrict the growth and dispersal of *X. subpileatus* in specific regions. Nevertheless, the reasons behind the localized increase in highly suitable areas remain unclear. These regions may possess unique ecological conditions or environmental factors that favor the species’ survival and expansion, which are absent elsewhere. For the period 1970–2000, North America and Asia contributed 23.41% and 20.03%, respectively, to the total current suitable area.

Furthermore, the predicted current potential distribution aligns closely with the documented occurrence records of *X. subpileatus* (Suppl. material 1: fig. S1), supporting the reliability of the model predictions.

### Future potential suitable regions

**Figure 4.**
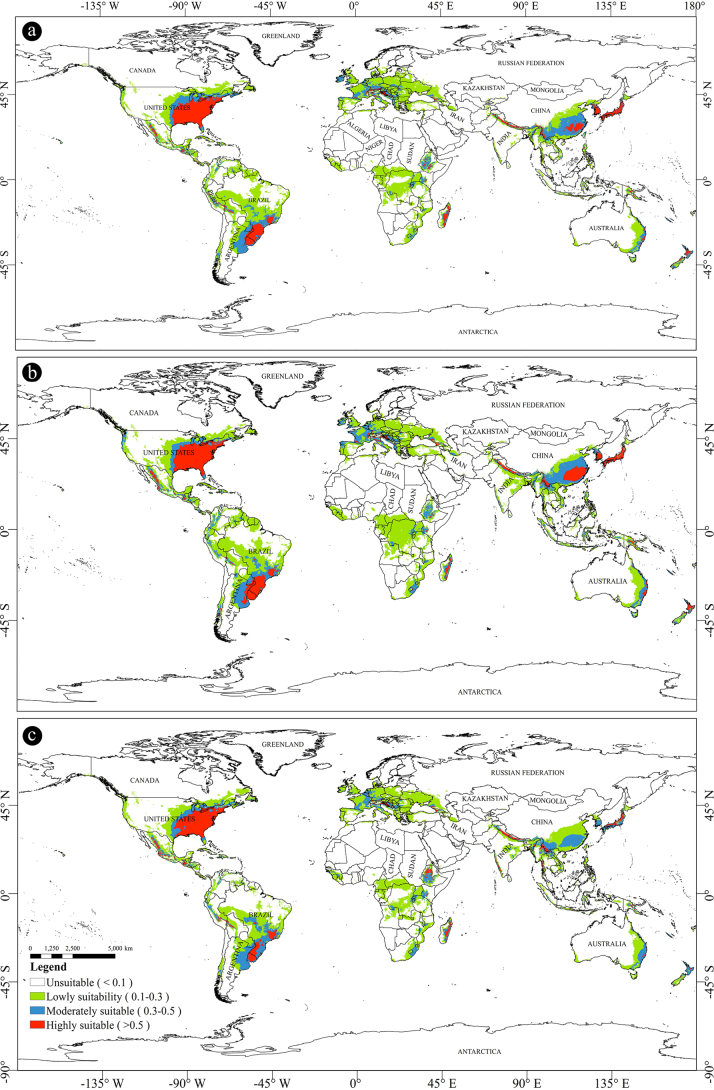
Predicted distribution of *X. subpileatus* under SSP2-4.5 scenarios in 2050s, 2070s and 2090s. **a**. 2050s (2041–2060); **b**. 2070s (2061–2080); **c**. 2090s (2081–2100). The source of map: Stimson 2023.

**Figure 5.**
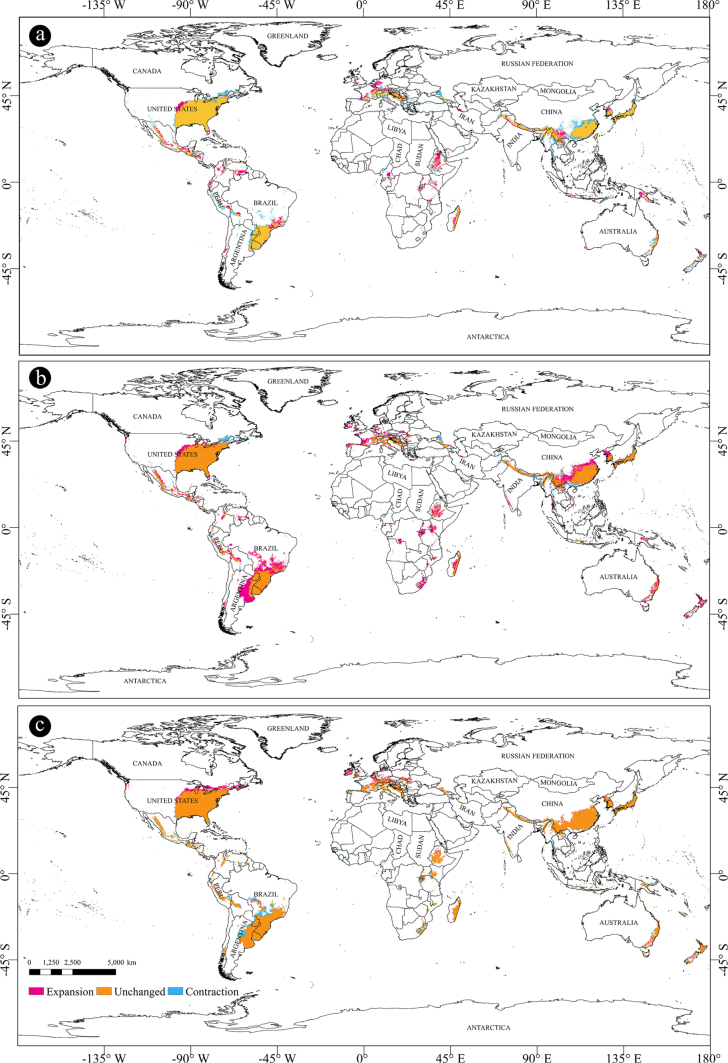
Changes of potential suitable areas of *X. subpileatus*. **a**. Mid Holocene–current; **b**. Current–2030s; **c**. 2030s–2050s. The source of map: Stimson 2023.

**Figure 6.**
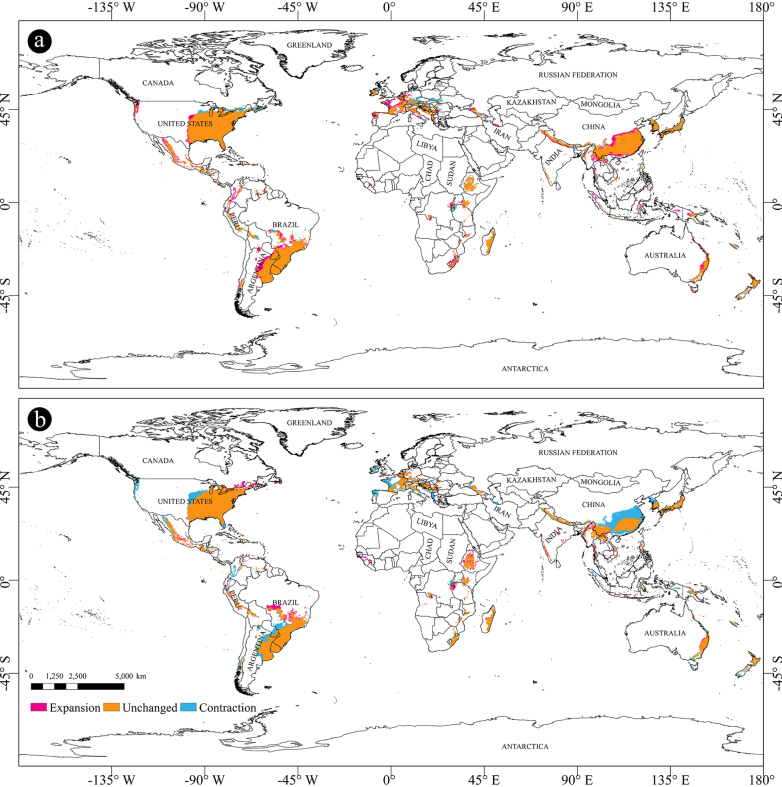
Changes of potential suitable areas of *X. subpileatus*. **a**. 2050s–2070s; **b**. 2070s–2090s. The source of map: Stimson 2023.

We further projected the potential distribution of *X. subpileatus* for four future periods: 2021–2040, 2041–2060, 2061–2080, and 2081–2100, respectively. Under the same climate scenario, the changing trends in potential habitats during these four periods displayed a high degree of similarity (Figs 4, 5, 6; Suppl. material 1: table SS1). The model predicted the most notable expansions of suitable habitats during the 2030s and 2070s, followed by a slight contraction in the 2050s. The predicted areas classified as lowly suitable, moderately suitable, highly suitable, and total suitable regions for these four time slices are summarized in Table 2.

When compared with the current distribution predictions, the results generated by the BCC-CSM2-MR model revealed a distinct spatial trend. Specifically, suitable habitats, including lowly, moderately, highly, and overall suitable areas, are projected to expand gradually toward coastal regions.

## Discussion

### Accuracy of model prediction

Among species distribution models (SDMs), the MaxEnt model plays a crucial role and is widely applied in ecology, biogeography, evolutionary biology, and species conservation (Araújo and Guisan 2006). In this study, the MaxEnt model was constructed using ten to twelve environmental variables and 232 occurrence records. The resulting AUC and TSS values demonstrated the model’s strong ability to generate precise simulation outcomes. The predictive accuracy of our model was higher than that reported in previous studies (Guo et al. 2019a; Bie et al. 2021; Chen et al. 2022). This improvement may be attributed to the quality control of species occurrence records and the careful screening of environmental variables. These two factors significantly influenced the accuracy of model predictions. In addition, the model parameters were specifically optimized in this study to further enhance predictive performance.

Overall, the predicted current potential distribution of *X. subpileatus* closely corresponds to its documented occurrence records. This finding provides valuable clues for identifying novel potential habitats. Future field investigations should prioritize these newly predicted regions, particularly those classified as highly suitable, to validate and refine the accuracy of the current model.

### Relationship between environmental variables and geographical distribution

The Jackknife test (Suppl. material 1: fig. S3) showed that the distribution of *X. subpileatus* is primarily constrained by mean annual temperature and annual precipitation. An annual precipitation range of 1185.11–1243.96 mm was found to be optimal for the growth of *X. subpileatus*, suggesting that the species favors relatively moist environments. The suitable annual mean temperature range is 9.40–14 °C (excluding the Mid-Holocene anomaly of 115.32 °C), indicating that the species is best adapted to subtropical and temperate climates. Our analysis further revealed that *X. subpileatus* relies heavily on water availability. For example, the precipitation of the driest quarter was between 242.48 and 368.22 mm, representing the widest ecological niche for this species. The distribution probability increases as the value of each key environmental variable rises toward its optimum, and then declines once the optimum value is exceeded. Climatic conditions are widely recognized as the dominant factors influencing the distribution of ectomycorrhizal fungi, saprotrophic fungi, plants, and animals (Walther et al. 2002; Wollan et al. 2008; Yuan et al. 2015; Zhao et al. 2022; Zhang et al. 2023). In particular, species-specific thresholds of temperature and precipitation strongly determine their geographic ranges.

Our results confirm that *X. subpileatus* is especially sensitive to moisture availability (bio12 and bio17–bio19). This underscores the importance of sufficient precipitation for its growth and development. The second most influential predictor identified in the MaxEnt analysis was annual mean temperature (bio1), which also plays a key role in determining the species’ distribution. Temperature affects physiological processes such as growth rate, development, and reproduction, thereby influencing the geographical range of ectomycorrhizal and saprotrophic fungi, as well as other organisms.

In conclusion, our findings emphasize the critical importance of climatic conditions, particularly temperature and moisture, in shaping the distribution patterns of *X. subpileatus*. These insights also provide a valuable reference for the conservation and management of fungi, plants, and animals under changing climate conditions.

### Analysis of changes in potential habitat areas

**Figure 7.**
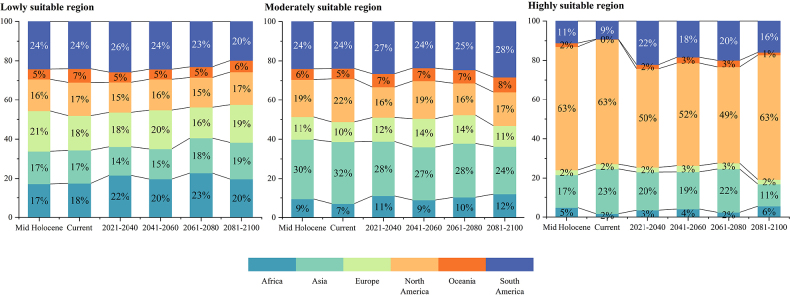
Areas and changes of habitats of different suitability for *X. subpileatus* at different times.

Generally, potential suitable habitats for many species tend to shift toward higher latitudes over time (Zhao et al. 2022). However, our results indicate a contrasting pattern. For *X. subpileatus*, the potential suitable areas migrated slightly toward lower latitudes, and the amplitude of this shift was minimal. The changes in potential suitable habitats of *X. subpileatus* across six time periods are presented below. It is important to note that this analysis focused only on moderately and highly suitable regions (Figs 5, 6). The species’ potential distribution spans six continents, excluding Antarctica, and the suitable areas on each continent have experienced dynamic changes over time (Fig. 7; Suppl. material 1: table SS1).

**Figure 8.**
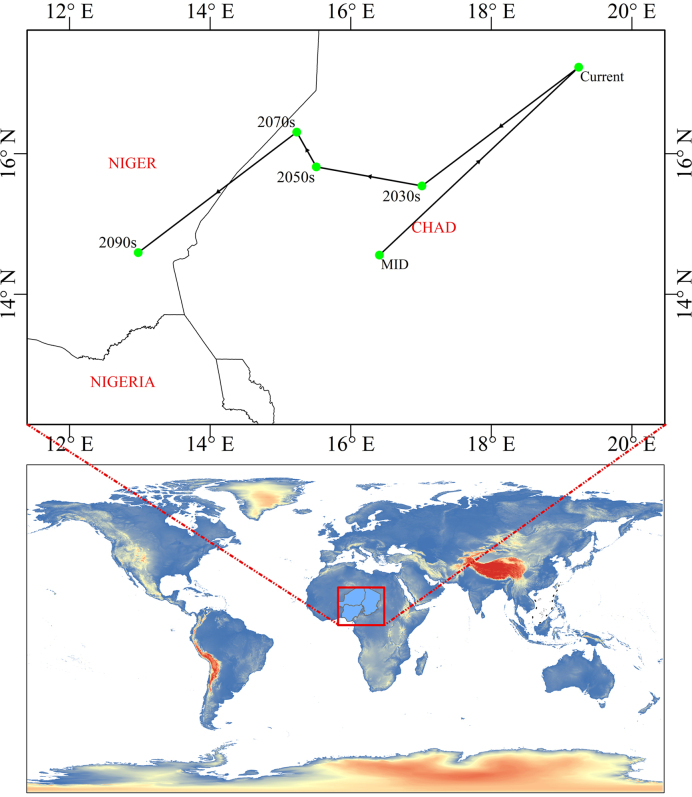
Centroid position shifts under different climatic conditions (Arrows symbolize the anticipated changes in both magnitude and orientation over time).

Because the spatial extent of suitable habitats for *X. subpileatus* is irregular, we used the distribution centroid to represent the central point of the predicted range. The centroid shift reflects changes in the spatial location of suitable habitats under different climate change scenarios. According to our analysis, the centroid during the Mid-Holocene period was located in Bahr el Gazel Nord, Bahr el Gazel, Chad, while under current climatic conditions it has shifted slightly northward to Borkou, Chad (Fig. 8; Table 3).

**Table 3.** Centroid coordinates of suitable area of *X. subpileatus* under different climatic conditions.

	MID	current	2030s	2050s	2070s	2090s
**latitude, longitude**	14.557148°N, 16.408769°E	17.229474°N, 19.243127°E	15.54065°N, 17.013633°E	15.811855°N, 15.510559°E	16.306803°N, 15.231747°E	14.592862°N, 12.97729°E

From the Mid-Holocene to the current period, the areas classified as lowly and moderately suitable increased, whereas the highly suitable regions decreased (Table 2; Suppl. material 1: table SS1). The overall extent of potential suitable habitats expanded significantly in Asia and North America, with the largest increases observed in the United States (24.82 × 10^4^ km^2^), Canada (23.11 × 10^4^ km^2^), and China (12.11 × 10^4^ km^2^). In contrast, considerable reductions occurred in regions of high habitat suitability across parts of Asia and adjacent areas of Africa. During the early to mid-Holocene, the geographical distribution of *Quercus* expanded progressively. However, beginning approximately 5000 years BP, this range underwent a marked contraction (van der Knaap et al. 2005; Guido et al. 2020; Hao et al. 2023). Obligate host-associated fungi such as *X. subpileatus* depend entirely on their hosts for survival and reproduction. Therefore, shifts in the spatial extent, abundance, or habitat continuity of Quercus populations are likely to have driven the observed decline of *X. subpileatus* in regions of high climatic suitability. These variations are likely linked to pronounced differences in temperature patterns during the Mid-Holocene, a phenomenon known as the Holocene temperature conundrum (Liu et al. 2014). This helps explain why the optimal annual mean temperature for the growth of *X. subpileatus* during the Mid-Holocene was anomalous compared to other periods. The slight reduction in highly suitable areas may also be attributed to the warmer and more humid climate prevailing during the Mid-Holocene relative to the current period.

From the current period to the 2030s, the potential distribution of *X. subpileatus* shows an overall expanding trend (Fig. 5b). Most of this expansion is concentrated in regions adjacent to the current suitable habitats, indicating a gradual spatial shift. At the same time, the species shows a tendency to migrate toward nearby coastal regions. The most substantial increases in total suitable areas are projected for South America, Africa, and Europe, with predicted expansions of 135.58%, 146.03%, and 117.7%, respectively. By the 2030s, the intensifying greenhouse effect is expected to cause significant increases in global temperature and precipitation. These warmer and more humid climatic conditions are likely to provide highly favorable environments for the growth and spread of *X. subpileatus*.

From the 2030s to the 2050s, the areas classified as highly and lowly suitable for *X. subpileatus* exhibited a slight contraction, while moderately suitable regions showed a small increase (Fig. 5c). The most notable reductions occurred in South America (87.97% of the 2030s value), Africa (85.82%), and Asia (98.34%). In contrast, the area of suitable habitats in Europe continued to expand. These findings suggest that, within a specific climatic threshold, elevated temperatures and increased precipitation resulting from greenhouse warming promote the growth of *X. subpileatus*. However, when global warming exceeds the species’ optimal tolerance range, varying degrees of contraction occur in its potential distribution.

From the 2050s to the 2070s, the potential distribution range of *X. subpileatus* expanded significantly once again (Fig. 6a). The expansion occurred mainly in Asia and Africa, where the total suitable area increased by 120.46% and 120.63%, respectively. Among individual countries, the most substantial increases were observed in India (58.39 × 10^4^ km^2^), the Democratic Republic of the Congo (40.28 × 10^4^ km^2^), and Indonesia (26.57 × 10^4^ km^2^). This expansion primarily occurred along coastlines and near major river systems, highlighting the role of hydrological conditions in shaping habitat suitability.

From the 2070s to the 2090s, a marked contraction in suitable habitats was observed across all suitability levels, including high, moderate, and low potential regions (Fig. 6b). The largest reductions were recorded in South America (79.57%), Africa (81.15%), and Asia (79.45%), compared to the corresponding 2070s values. Additionally, Fig. 6 illustrates significant changes in China, where the areas classified as highly and moderately suitable decreased by 45.92% relative to the 2070s. This sharp decline underscores the species’ sensitivity to warming beyond its optimal ecological thresholds.

## Conclusion

The MaxEnt model indicated that the suitable habitat for *X. subpileatus* initially expanded and subsequently contracted during the Mid-Holocene, the current period (1970–2000), and the future periods (2021–2040, 2041–2060, 2061–2080, and 2081–2100). Annual precipitation and annual mean temperature were identified as the principal environmental variables constraining the species’ spatial distribution. These factors play a critical role in determining habitat suitability by influencing species survival, growth, and reproduction. The primary distribution area is projected to remain in North America, particularly in the eastern United States, where diverse ecosystems and climatic conditions provide favorable habitats for *X. subpileatus*. However, climate change is expected to modify these distribution patterns, which may pose challenges to species conservation. Crucially, the occurrence of the species at a given site depends not only on favorable climatic conditions but also—equally importantly—on the availability of suitable host trees. Overall, the findings of this study provide valuable insights into the potential impacts of climate change on species habitat and distribution, highlighting the need for adaptive conservation strategies to ensure the long-term survival of *X. subpileatus*.

## Additional information

### Artificial Intelligence (AI) use

The authors accept full responsibility for the content of the manuscript, including the disclosure of any use of AI.

No AI tools were used in the preparation of this manuscript.

### Author ORCIDs

Y. Xu https://orcid.org/0000-0002-8935-4432

A. Niu https://orcid.org/0000-0002-6533-0807

J. Cao https://orcid.org/0000-0003-4724-8145

R. Fan https://orcid.org/0000-0002-9497-8274

S. He https://orcid.org/0000-0003-4702-3034

### Data availability

All of the data that support the findings of this study are available in the main text or Supplementary Information.

## Supplementary materials

Supplementary material 1 Supplementary figures and tables Data type docx https://binary.pensoft.net/file/1660344This dataset is made available under the Open Database License (http://opendatacommons.org/licenses/odbl/1.0/). The Open Database License (ODbL) is a license agreement intended to allow users to freely share, modify, and use this Dataset while maintaining this same freedom for others, provided that the original source and author(s) are credited. Yunlin Xu, Anyi Niu, Jiaxi Cao, Rong Fan, Shuanghui He
