# Supplementary material for: Impacts of climatic factors on the global spatiotemporal distribution patterns of Xylobolus subpileatus (Stereaceae, Russulales)
Source: MycoKeys. 2026 May 28;133:127–48. doi: 10.3897/mycokeys.133.190063 (PMC13237564; doi:10.3897/mycokeys.133.190063)
Supplement: Supplementary material 1 — Supplementary figures and tables [file mycokeys-133-127-s001.docx]

**Supplementary Materials**

**1. Supplementary Figures and Table**

**1.1 Supplementary Figures**

**
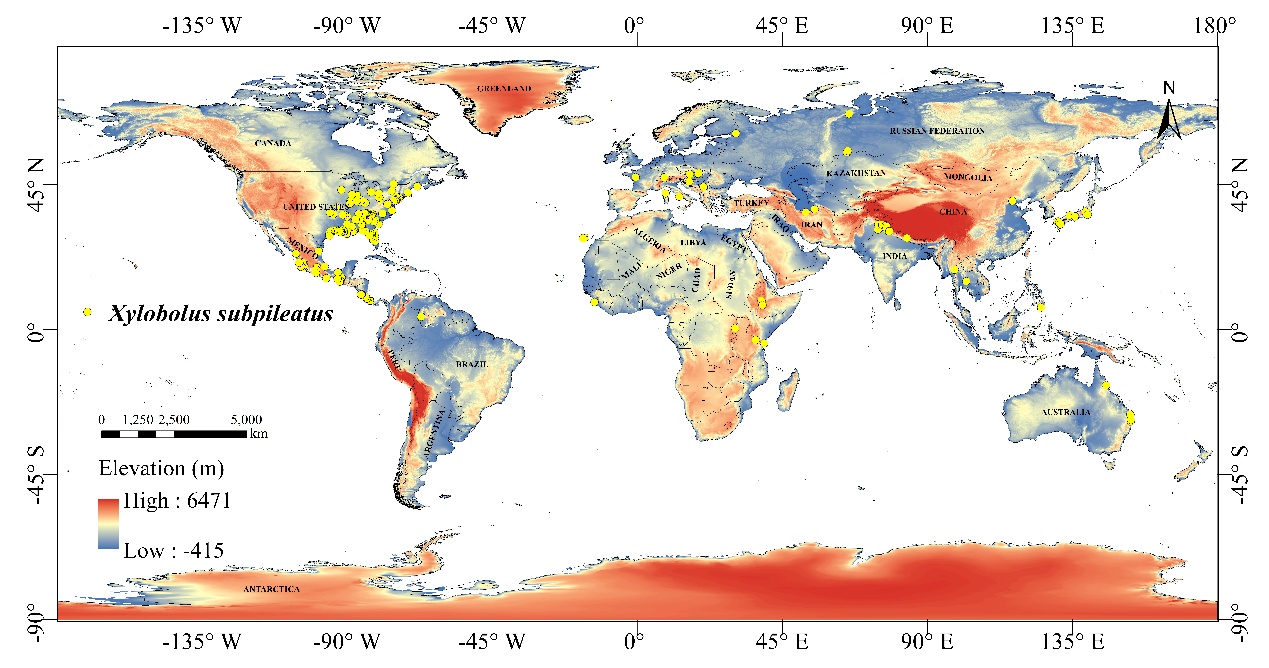
**

**Figure S1** The occurrence data (232 points) of *Xylobolus subpileatus*

**
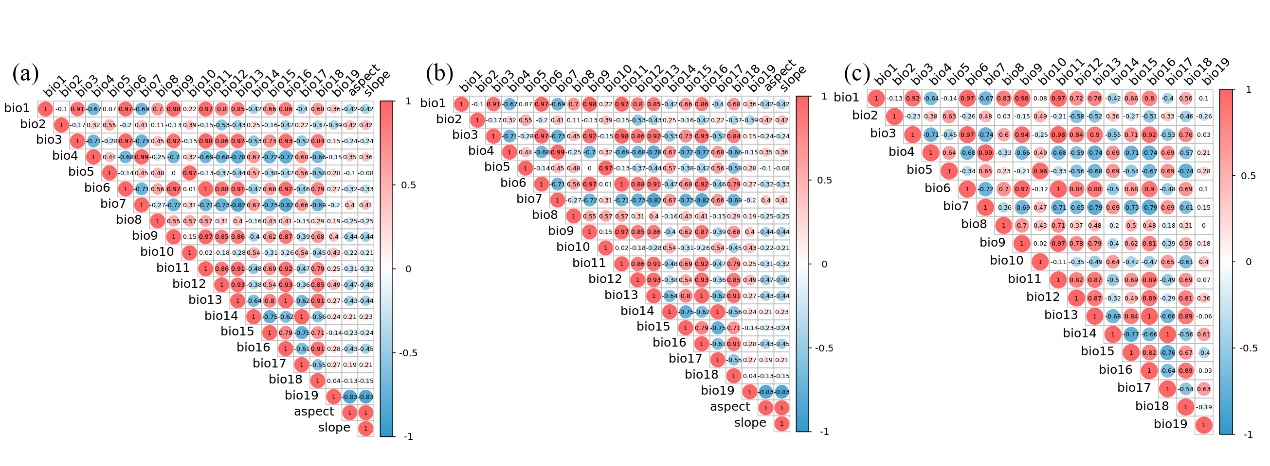
**

**Figure S2** Correlation of environmental variables for modeling the potential distribution of *Xylobolus subpileatus*. (a) Mid Holocene (b) current (c) future


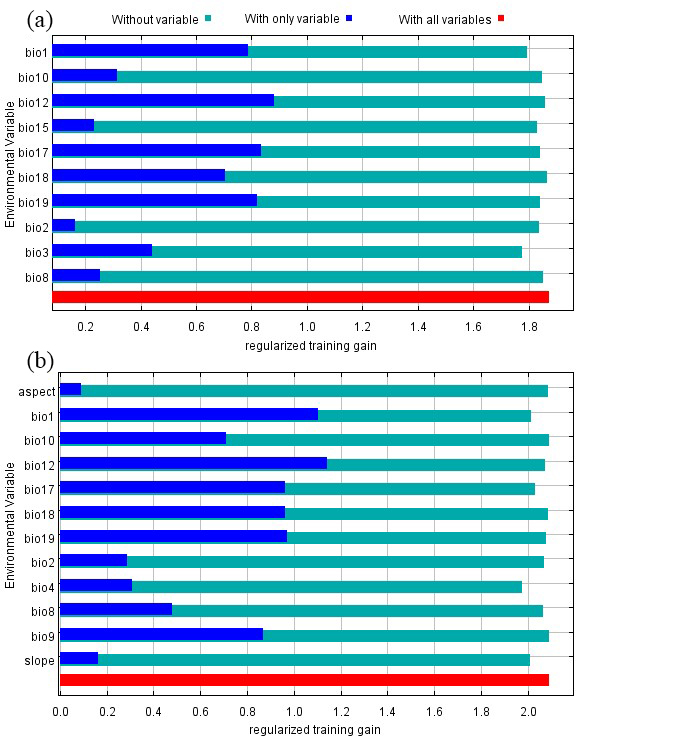


**Figure S3** Importance of major environmental variables on distribution of *Xylobolus subpileatus* by Jackknife test (a) Mid Holocene (b) current.

**1.2 Supplementary Table**

**Table S1** Distribution area with different suitable index of *Xylobolus subpileatus* on global scale (× 10^4^ km^2^)

| **Region** | **MID** | | | **Current** | | | **2021-2040** | | | **2041-2060** | | |
| --- | --- | --- | --- | --- | --- | --- | --- | --- | --- | --- | --- | --- |
|  | Lowly | Moderately | Highly | Lowly | Moderately | Highly | Lowly | Moderately | Highly | Lowly | Moderately | Highly |
| **Asia** | 214.83 | 96.59 | 42.97 | 297.84 | 109.15 | 25.32 | 318.94 | 114.9 | 20.75 | 301.62 | 80.82 | 34.15 |
| **Europe** | 196.36 | 21.44 | 2.42 | 275.71 | 32.78 | 3.26 | 355.52 | 32.8 | 4.69 | 321.69 | 34.96 | 4.93 |
| **North America** | 187.44 | 112.64 | 188.01 | 235.02 | 124.63 | 192.59 | 239.2 | 97.94 | 216.44 | 246.84 | 101.11 | 214.13 |
| **South America** | 198.67 | 54.8 | 3.8 | 314.44 | 85.8 | 8.43 | 328.13 | 143 | 19.84 | 301.97 | 141.67 | 9.6 |
| **Africa** | 99.43 | 22.38 | 15.97 | 131.86 | 20.76 | 5.95 | 227.01 | 44.93 | 14.17 | 204.45 | 40.62 | 11.89 |
| **Oceania** | 48.36 | 7.35 | 0.66 | 112.02 | 12.89 | 0.64 | 70.65 | 12.95 | 0.71 | 72.95 | 13.77 | 0.23 |
| **Global** | 945.09 | 315.2 | 253.83 | 1366.89 | 386.01 | 236.19 | 1539.45 | 446.52 | 276.6 | 1449.52 | 412.95 | 274.93 |

**Table S2** Centroid coordinates of suitable area of *X. subpileatus* under different climatic conditions

|  | MID | current | 2030s | 2050s | 2070s | 2090s |
| --- | --- | --- | --- | --- | --- | --- |
| longitude | 16.40877 | 19.24313 | 17.01363 | 15.51056 | 15.23175 | 12.97729 |
| latitude | 14.55715 | 17.22947 | 15.54065 | 15.81186 | 16.3068 | 14.59286 |
